# Emergence of Polymeric Material Utilising Sustainable Radiation Curable Palm Oil-Based Products for Advanced Technology Applications

**DOI:** 10.3390/polym13111865

**Published:** 2021-06-04

**Authors:** Rida Tajau, Rosiah Rohani, Mohd Sofian Alias, Nurul Huda Mudri, Khairul Azhar Abdul Halim, Mohd Hamzah Harun, Naurah Mat Isa, Rosley Che Ismail, Sharilla Muhammad Faisal, Marina Talib, Muhammad Rawi Mohamed Zin, Izzati Izni Yusoff, Nadiah Khairul Zaman, Iqma Asyila Ilias

**Affiliations:** 1 Department of Chemical & Process Engineering, Faculty of Engineering and Built Environment, Universiti Kebangsaan Malaysia, UKM Bangi, Selangor 43600, Malaysia; nurul.izzati.izni@gmail.com (I.I.Y.); nadiahkz@gmail.com (N.K.Z.); p111889@siswa.ukm.edu.my (I.A.I.); 2 Radiation Processing Technology Division, Malaysian Nuclear Agency, Bangi, Kajang, Selangor 43000, Malaysia; sofian@nuclearmalaysia.gov.my (M.S.A.); nurul_huda@nuclearmalaysia.gov.my (N.H.M.); azharhalim@nuclearmalaysia.gov.my (K.A.A.H.); hamzah@nuclearmalaysia.gov.my (M.H.H.); naurah@nuclearmalaysia.gov.my (N.M.I.); rosley@nuclearmalaysia.gov.my (R.C.I.); sharilla@nuclearmalaysia.gov.my (S.M.F.); marina@nuclearmalaysia.gov.my (M.T.); muhammad_rawi@nuclearmalaysia.gov.my (M.R.M.Z.)

**Keywords:** biomaterial, palm oil, radiation curing, radiation processing, surface coatings, VOC

## Abstract

In countries that are rich with oil palm, the use of palm oil to produce bio-based acrylates and polyol can be the most eminent raw materials used for developing new and advanced natural polymeric materials involving radiation technique, like coating resins, nanoparticles, scaffold, nanocomposites, and lithography for different branches of the industry. The presence of hydrocarbon chains, carbon double bonds, and ester bonds in palm oil allows it to open up the possibility of fine-tuning its unique structures in the development of novel materials. Cross-linking, reversible addition-fragmentation chain transfer (RAFT), polymerization, grafting, and degradation are among the radiation mechanisms triggered by gamma, electron beam, ultraviolet, or laser irradiation sources. These radiation techniques are widely used in the development of polymeric materials because they are considered as the most versatile, inexpensive, easy, and effective methods. Therefore, this review summarized and emphasized on several recent studies that have reported on emerging radiation processing technologies for the production of radiation curable palm oil-based polymeric materials with a promising future in certain industries and biomedical applications. This review also discusses the rich potential of biopolymeric materials for advanced technology applications.

## 1. Introduction

The current evolution of radiation curable bio-based materials from various oil sources using radiation processing technologies has attracted the interest of scientists and technologists for developing novel biomaterials for diverse uses [1,2,3,4,5,6]. Synthetic polymers used in the processing of biomaterials have certain disadvantages, such as being highly toxic, non-biodegradable, and expensive. On the other hand, natural polymeric materials are simple to synthesise, low-cost, biodegradable, biocompatible, non-immunogenic, non-toxic, and biologically safety [7].

Natural vegetable oils and fatty acids that are mostly edible (e.g., palm oil, soya oil, jojoba oil, rapeseed oil, olive oil, and canola oil) have been widely used in the development of polymeric biomaterials, such as in coatings, lubricants, agrochemicals, and plasticisers [8,9,10]. The presence of carbon double bonding (-C=C-) in vegetable oils makes it easy to function with reactive groups, such as epoxy, hydroxyl, carboxyl, and acrylate, which allows the preparation of polyurethane, elastomers, plastics, and pressure-sensitive adhesives (PSA) [10,11,12]. Therefore, it has given attractive benefits to vegetable oils, unlike the toxic, costly, and non-environmentally friendly feedstocks taken from petroleum. Palm oil is among the most attractive renewable alternatives, and palm oil’s inherent biodegradability is also a desirable feature in the context of rising environmental concerns compared to petroleum-based products concerning to minimising production costs and maximising inputs and outputs, such as food supply, water management, recovery of energy and products, and waste treatment [13]. Indonesia, Malaysia, and Thailand being the largest producers of palm oil in ASEAN countries will contribute to a significant field of palm oil industry for the oleochemical market.

**Figure 1:**
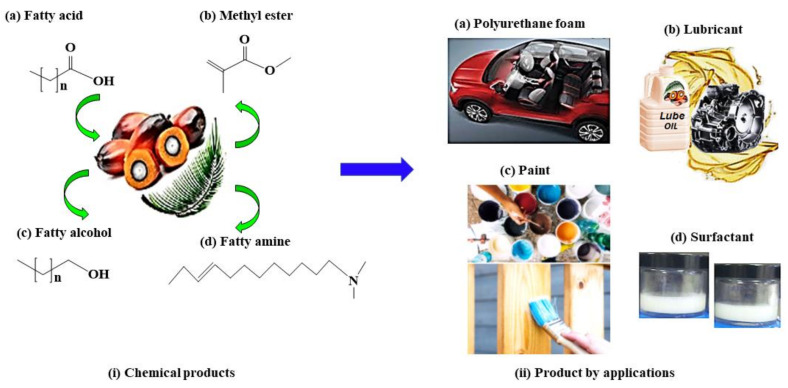
Palm oil used in oleochemical industry: (**i**) chemical products (**ii**) and formulated products by applications.

Fatty acids, methyl esters, fatty alcohols, and fatty amines (Figure 1i) derived from natural oil are essential building blocks for the production of polyurethanes, surfactants, lubricants, and other products in the oleochemical industry (Figure 1ii). Therefore, the palm oil industry plays a crucial role in contributing to environmental sustainability and helps to reduce poverty of millions of palm oil workers and their families. Besides, these based sources have essential chemical compositions and ingredients for the development of food and non-food products.

The development of radiation curable palm oil-based biomaterials seems to be one of the potential future applications of palm oil products, as this novel material has the potential to contribute positively to the palm oil industry. Expansion of the existing applications of palm oil products into the pharmaceutical industry is of interest because palm oil is an edible vegetable oil with excellent physicochemical properties, such as ability to modify chemical composition for biomaterials development. Likewise, other palm oil-based products, such as refined-bleached-deodorised (RBD) palm oil, palm oil, and palm stearin are abundantly available in Malaysia. They are known to be economical, sustainable, and environmentally biodegradable [3]. Palm oil is consumed worldwide as food products and has positive health properties, including antioxidants, anti-cancer, cholesterol-reducing effects, and is biologically active as pro-vitamin [14,15,16]. These palm oil-based products are promising prospects for manufacturing biomaterials that become alternative products to other polymers from synthetic/chemical-based.

Meanwhile, radiation processing technology has been reportedly used to modify the properties of materials in either bulk or surface structures with the aim to produce novel materials and products that pose desirable structures and properties [17,18,19,20]. Radiation processing can be properly controlled based on its delivery dosage, penetration depth, time of exposure, and raw material(s) used. This radiation method is known for its simplicity, rapid process, easy to control, and efficient for sterilisation. It is also a solvent-free process with low or no volatile organic compounds (VOC) and require fast-curing at room temperature only. These advantages allow the synthesis to be performed at low energy and require minimal working space. Radiation is also a particular energy source which can cause chemical reactions at any temperature (including ambient temperature), pressure, phase (gas, liquid or solid), and without assessment of a catalyst [21].

The general advantages of radiation processing techniques are simple process control, cost-effective, and safe for the environment. Therefore, these techniques are commonly used for modification of polymeric materials, polymer cross-linking, monomer and polymer curing, polymer grafting, and polymer degradation. These techniques are being utilised to develop new materials and advanced products, for example, nanomaterials and lithography (nanogels, metal nanoparticles, carbon nanotubes, nanocomposites), for different branches of industry [22,23]. Besides, radiation processing using gamma, electron beam, and UV sources have being explored and used for preservation and protecting cultural heritage artefacts, including desinsection, disinfection, restoration, and conservation to prevent further decay and damage [24].

Due to numerous benefits, this review is formulated to focus on the radiation curable palm oil-based materials prepared from radiation processing technologies. Critical discussions are presented in this review, especially on the palm oil-based polymeric sources, such as acrylate, polyol, and polyurethane, their properties and present use. The fundamental of radiation engineering for the production of biomaterials and their possible usage in various present and future applications are also discussed. This review is specifically aimed to strengthen the biomaterials benefits and potential applications in comparison to the well-known synthetic polymers. It is also especially aimed to reinforce radiation processing technology to be used for converting radiation curable palm oil-based products into novel biomaterials.

## 2. Fundamentals of Radiation Processing in Polymeric Biomaterial Design

Radiation-induced material changes are dependent on the source and form of the radiation and the energy deposited [25]. Radiation is classified into two primary classifications, which are non-ionising and ionising radiations. Ionising radiation is extraordinarily energetic and has sufficient energy as particles and electromagnetic waves to ionise a material molecule. Non-ionising radiation is the term used for radiation in the electromagnetic waves, where insufficient energy is required to induce the ionisation that requires a photo-initiator. The method of energy deposition relies on the source of radiation, such as particulate radiation (including electrons, positrons, protons, neutrons, and ions) or electromagnetic radiation (including infrared, visible and ultraviolet radiation, and X-rays and gamma rays).

**Figure 2:**
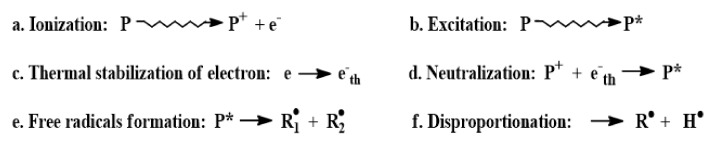
Primary processes occurring of polymer in ionising irradiation. * P is a polymer and R^●^ is a radical. Source: Adapted Vasile and Butnaru [26].

Polymer irradiation is an industrial process that is very useful. It is an alternative to more conventional chemical processes in which the method may induce or modify the properties of the material. Primary interactions of polymers (either natural or synthetic polymers) with ionising radiation include ionisation, excitation of molecules and atoms, stabilisation of electrons by the generation of hot electrons, ion neutralisation, and free radical formation. These result in the formation of excited states of reactive intermediates, free radicals, ions, atoms, and molecules, as shown in Figure 2.

**Figure 3:**
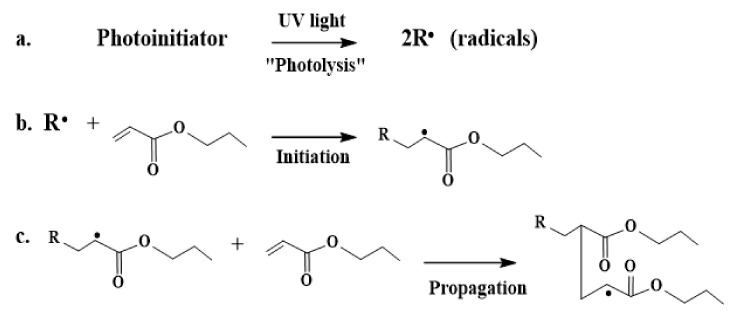
Primary mechanism of free radical polymerisation under UV radiation of (**a**) photolysis, (**b**) initiation, and (**c**) propagation.

In non-ionising radiation, such as UV radiation, free radicals are produced typically by hydrogen abstraction when the photo-initiator is exposed to UV light (Figure 3a). Radicals formed during the initiation step (Figure 3b) propagate further via a free radical process (Figure 3c). Figure 3 demonstrates the mechanism of the reaction of the free radical formation by photo-initiators.

**Figure 4:**
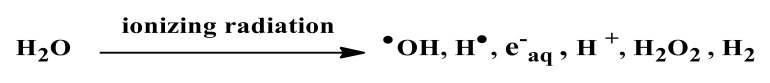
Primary process of radiolysis of water under irradiation. Source: Adapted Rosiak and Ulanski [27].

In the case of polymer in a dilute aqueous solution, the primary reaction involves the radiolysis of water, where water molecules absorb the energy of ionising radiation. This reaction results in the creation of reactive intermediates: hydroxyl radicals (^●^OH), hydrogen atoms (H^●^), hydrated electrons (e^−^_aq_), hydrogen ion (H^+^), hydrogen peroxide (H_2_O_2_), and hydrogen molecule (H_2_) (Figure 4). 

**Figure 5:**
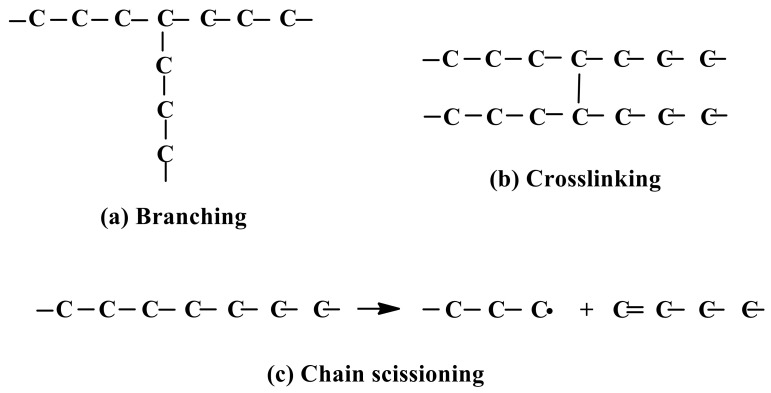
Scheme of chain branching, cross-linking, and chain scissioning.

Furthermore, the subsequent interactions include either the transfer of electrons; the abstraction of hydrogen atoms; or the addition of C=C, C=N, C=S, aromatic rings, or an electron-rich functional group. This process contributes to the creation of radical sites in the polymer chain. It also allows the macroradicals to follow several different pathways of reaction, such as chain scission, hydrogen transfer, inter- or intramolecular recombination, and disproportion, which ultimately result in the formation of oxidised products, branching, grafts, scission/degradation, or cross-linking of the main chains of polymers, as shown in Figure 5 [28].

Chain branching forms a growing chain of polymers from the original chain of polymers, cross-linking forms an insoluble three-dimensional network of polymers, whereas degradation or scission involves the breaking of chemical bonds on the polymer backbone. These schemes of chains can be seen in Figure 5. Chain branching and cross-linking processes increase the polymeric molecular weight, while degradation or scission causes the initial molecular weight to be reduced. Overall, radiation processing technology is widely used to modify the material properties, and novel materials with desirable structures and properties can be produced as well.

## 3. Radiation Curable Palm Oil-Based Materials

**Figure 6:**
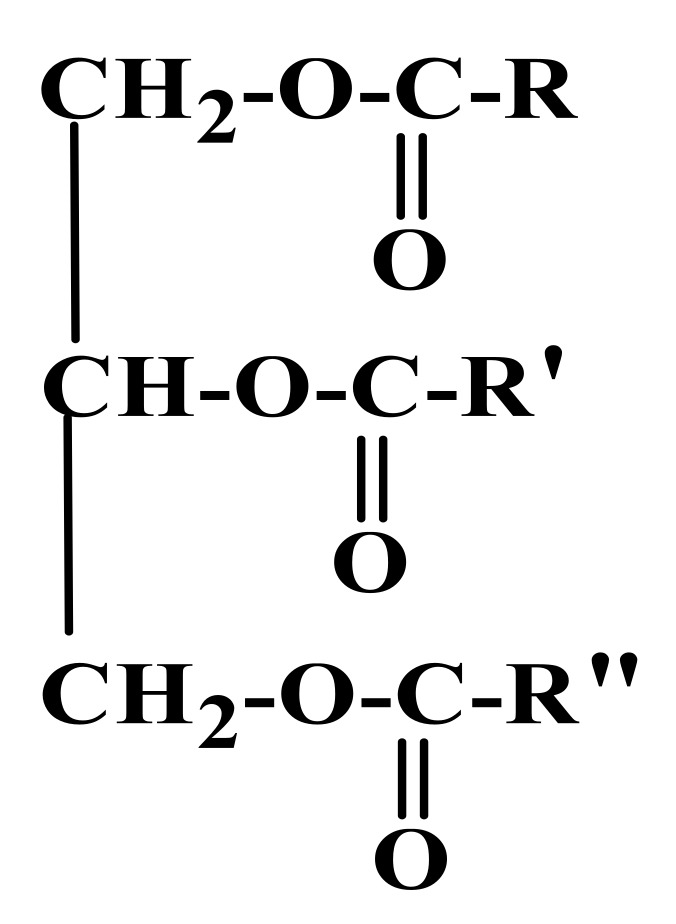
General chemical structure of palm oil (-R can be represented by palmitic, stearic, myristic, oleic, and linoleic).

**Figure 7:**
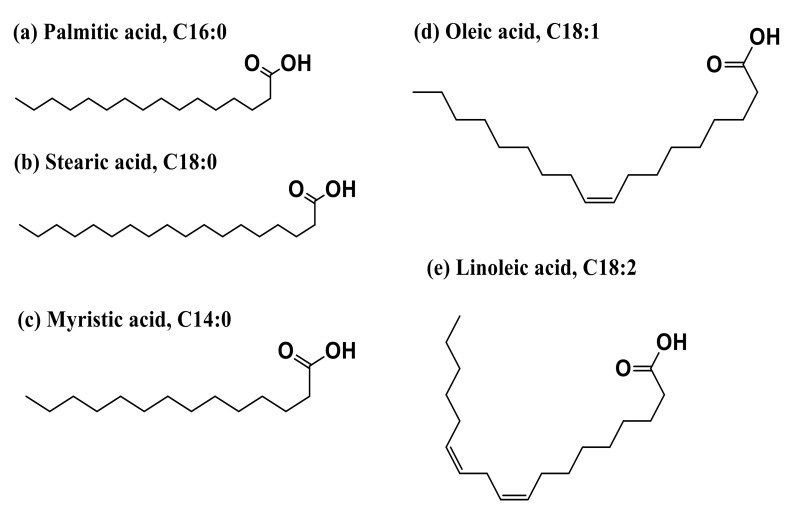
Chemical fatty acids structure in palm oil (C-C: saturation; C=C: unsaturation). It has high levels of saturated fatty acids, about 50% of which include palmitic (44.3%), stearic (4.6%), and myristic (1.0%). It also contains around 38.7% of oleic acid monounsaturated fatty acid and 10.5% of linoleic acid polyunsaturated fatty acid. Source: Adapted from Amir Reza, Suraya, and Azmi [30].

Bio-based materials produced from vegetable oils via radiation curable process have received great attention for various applications in recent years. Palm oil, in particular, is directly obtained from the red-orange mesocarp of oil palm *Elaeis guineensis* fruit. It is edible and used worldwide as the main ingredient in many food products [29]. Palm oil has a complex mixture of triglyceride compounds, which can be seen in Figure 6. It is composed of fatty acids, including palmitic, stearic, myristic, oleic, and linoleic acids (Figure 7). 

**Figure 8:**
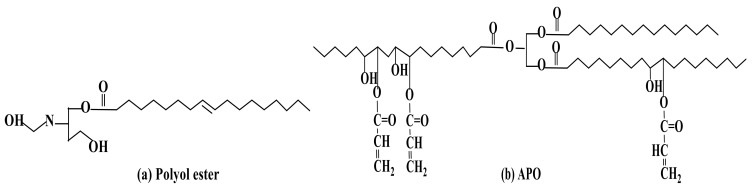
The general chemical structure and products of (**a**) polyol ester and (**b**) APO.

In the recent past, palm oil has gained significant interest and been extensively researched for nutritional, medicinal, and chemical applications, such as antioxidant activities, cholesterol-lowering benefits, anti-cancer effects, protection against atherosclerosis, emulsion and cosmetic formulations, and non-food products [31]. The palm oil products, such as polyol [32] and epoxidised palm oil acrylate (EPOLA^®^) [33], which are referred as Acrylated Palm Olein (APO) [34], have received considerable interest in many applications, such as surface finishing, packaging, automotive, and biomaterial (Figure 8). These products have shown remarkable physicochemical properties, as reported in many studies, which have been summarised in Table 1. The molecular weights and functional groups of polyol ester and APO play a vital role in the development and determination of the properties of the new products. They possess low molecular weight of below 5500 Daltons and hydrocarbon chains with ester compounds that could play a big role in hydrolytic degradation process. This inherent property could give additional biodegradability of APO and polyol ester compared to petrochemical-based polymers or synthetic polymers. Besides, their chemical structures, such as carbon double bonds (-C=C-) and hydroxyl groups (-OH), are also essentially useful in fine-tuning and developing new products with unique properties.

**Figure 9:**
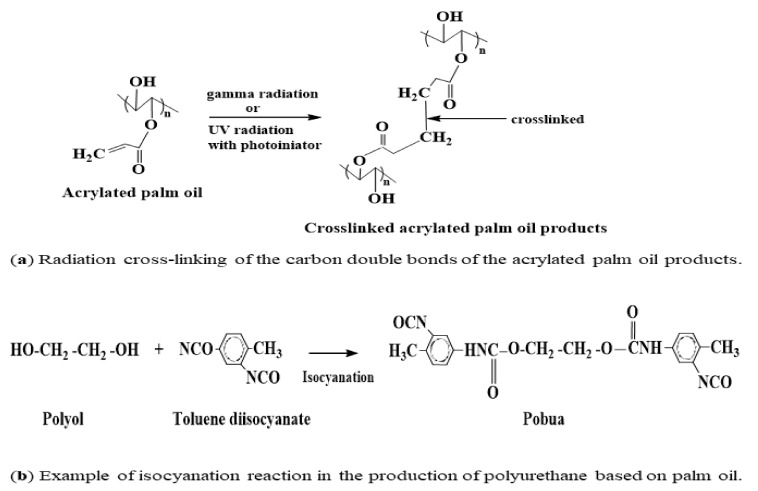
Application of acrylated palm oil and polyol in (**a**) radiation polymerisation of EPOLA^®^ or APO and (**b**) isocyanation of polyol in polyurethane production.

Many studies have demonstrated that EPOLA^®^, APO and polyol have shown promising characteristics in applications for radiation processing technology. For example, the presence of carbon double bonds in EPOLA^®^ and/or APO are reactive sites to promote modification of materials through radiation technology, such as via UV and gamma radiation. Upon exposure to radiation, these bonds then form cross-linked structure that can give enormous properties to new products (refer to Figure 9a). Moreover, the polyol reactive hydroxyl group provides a medium for chemical modification and surface functionalisation. Palm oil-based polyols with functional hydroxyl groups, for instance, have been widely used to modify polyurethane products (the image can be seen in Figure 9b) [35].

**Figure 10:**
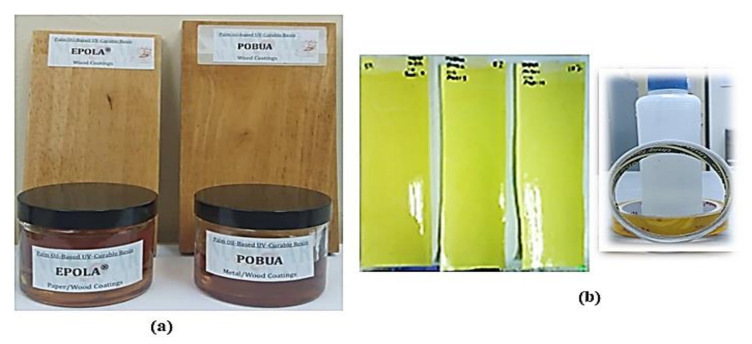
Radiation-curable of palm oil-based products with UV curing. (**a**) Acrylated and polyurethane resins for wood coating; (**b**) Polyurethane resins for PSA. Source: Unpublished data.

Concurrently, Figure 10 shows the recent applications of advanced radiation curing technology utilising palm oil-based materials. EPOLA^®^ can been seen to produce radiation-curable products, such as coatings (refer to Figure 10a) [36], pressure-sensitive adhesives (refer to Figure 10b) [37], printing inks and overprint varnishes [38], while APO is used to develop drug delivery systems. Meanwhile, polyols based on palm oil are used to produce polyurethane for the manufacture of foams, elastomers, plastics, and cast and injection-moulded components. These show that radiation-curable products based on palm oil have many usages for different sectors, such as agricultural, military, automobile, manufacturing and medical [10,37]. Next, descriptions of the application of radiation processing for the production of radiation-cured palm oil products for usage in various future developments will be addressed in the following section.

## 4. Current Application of Radiation Curable Palm Oil-Based Polymeric Materials

### 4.1. Surface Coating

The production of EPOLA^®^ and polyol-type polymers is believed to ultimately eliminate a large portion of the supply of petroleum-based polymers. Since they are derived from natural resources, their usages seem to be potentially cost-effective for developing natural polymers compared to petroleum-based polymers. This is because palm oil is a sustainable resource and has a reliable manufacturing line for production of raw materials. Besides, a majority of acrylic and polyol-type polymers currently in the market are petrochemical-based. Therefore, these innovations could boost industries that utilise polymeric coatings to focus on using sustainable commodities, such as from palm oil as potentially cheaper feedstocks, rather than petrochemical-based substances that are usually costly and non-sustainable [3].

Radiation-curable materials, such as EPOLA^®^, polyol, and polyurethane have shown inherent properties for application in coating industries, which include its physicochemical, environment, and chemical resistance, thermal and mechanical properties [2,3,39,40,41,42,43,44,45,46]. Thermal stability and mechanical properties are mainly referred in the characterisation of the surface coating’s thermal durability and reliability. Negligence in considering these properties would result in products cracking, delamination of the layers, and products deformation. These properties play a critical role in controlling the deposited coating elements in order to ensure that the quality of the substrates is maintained.

The thermal and mechanical properties of EPOLA^®^ and polyol-based products and their most promising applications upon exposure to UV irradiation are summarised in Table 2. Based on Table 2, EPOLA^®^ is seen to be widely used in various types of applications, such as surface coating resin, overprint varnish, adhesive, inks, and thermoplastic production. In contrast, polyol is used in miscellaneous polyurethane materials, such as ceiling panels, flora foams, polyurethane sheets, wall panels, roof insulators, laminated boards, adhesives, coatings, cushion, flexible foams, and automotive parts [3,47,48,49,50]. Materials based on EPOLA^®^ and polyol are produced from products based on palm oil, such as epoxy palm olein and oleic acid. *They are specially developed as radiation curable resin for making surface coating products.* Next, the hardness test results presented in Table 2 shows that the acrylic and polyurethane resins based on EPOLA^®^ have nearly identical ranges of deformation resistance properties as compared to polyol-based polyurethane. Meanwhile, the polyol-based polyurethane also displayed lower temperature degradation as compared to the resins-based EPOLA^®^. Different thermal and mechanical properties presented in Table 2 were attributed to various processing and formulation methods for ingredients, photo-initiators, and absorbed radiation dose of the resin products developed from EPOLA^®^ and polyol.

The substitution of radiation-curable resins from palm oil is known to substitute petrochemical-based resins with multifunctional developmental coating resins. Multifunctional coatings can provide additional durability advantages, including surface resistance to mildew, fungus, bacteria, and microorganisms; anti-fouling without metals; super-hydrophobicity; self-healing anti-corrosion; self-healing scratch repair; and thermochromic. Such features can open up modern, evolving applications of radiation-curable palm oil-based resin, such as in automobile, aerospace, and telecommunication devices; military and heavy-duty equipment; and agricultural machinery. The radiation-curable palm oil-based materials also gained strong attraction due to their variety of use, not only in coating, over print varnishes, ink, and adhesive industrial applications but also in the medical industry in particular to fulfil the needs for the production of biomaterial from natural resources relative to petrochemical source materials that tend to cause toxicity. Therefore, to ensure that the safety and effectiveness of these biomaterials are acceptable for the delivery of drugs throughout the human body, the production of a drug delivery system using radiation-curable palm oil-based materials was foreseen relevant.

### 4.2. Drug Delivery Systems

In recent years, palm oil has received considerable research interest in the delivery of drugs. This is supported by the summary presented in Table 3 that outlines the latest production of micro- or nano-scale palm oil-based products used in drug delivery. The summary shows that the standard techniques for the development of the micro- or nano-scale palm oil-based products include double casting, emulsion, micro-emulsion, nano-emulsion, high pressure homogenisation (HPH), particles from gas saturated solutions (PGSS), free thaw, thin-film hydration, laser ablation, and radiation techniques to be used in drug carrier field. Table 3 also summarises the current strategies for designing pharmaceutical products that utilise palm oil-based materials using different methods.

The results summarised in Table 3 also shows that palm oil-based products developed using different techniques stated above are capable of producing less than 500 nm of particle size. The produced particles in this range of size are found to be highly useful for oral, transdermal, topical, parenteral, pulmonary, antibacterial, and drug delivery systems, especially for breast cancer. Microemulsion is found as the most commonly used technique in manufacturing pharmaceutical products due to its low-cost, simplicity, efficient method, and affordability. On the other hand, the combination of microemulsion and radiation processing techniques has been announced recently as a promising strategy for creating a new type of drug carrier [17,27,28,56,57,58]. Using these combined techniques, the three-dimensional (3D) network structure and nanoparticles from the palm oil-based products such as APO and polyol ester has been developed.

**Figure 11:**
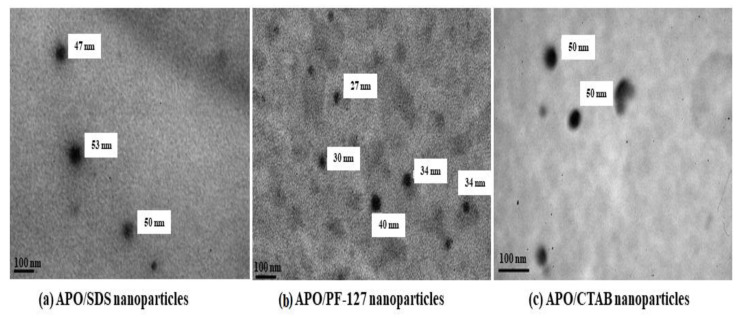
TEM images of the gamma-irradiated APO nanoparticles at different surfactants’ microemulsion system. APO composition of 0.18% in the microemulsion produces a variety of sizes of nanoparticles, such as 47–53 nm in the sodium dodecyl sulphate (SDS), 27–40 nm in the Pluronic-127 (PF-127), and 50 nm in the cetyltrimethylammonium bromide (CTAB) microemulsions. Source: Unpublished data.

**Figure 12:**
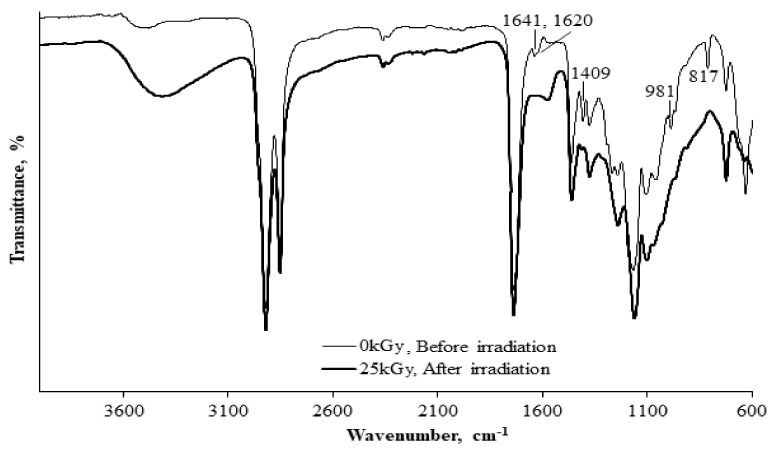
FTIR spectra of APO/CTAB matrix (**a**) non-irradiated; (**b**) irradiated at 25 kGy.

The development of drug delivery systems via radiation of materials, such as polymeric micelle and nanoparticles has been preliminary initiated using APO. Gamma-induced cross-linking has been successfully conducted on different types of APO’s microemulsion for developing the micro/nanoparticles [72]. These nanoparticles were dispersed in aqueous media at the critical micelle concentration of the surfactant and exposed to gamma irradiation to generate the nanoparticles. Figure 11 shows the TEM images of APO nanoparticles produced via gamma irradiation. The use of gamma-ray irradiation induces the hydroxyl radical forming in water to promote the double bond of acrylate carbon in APO that facilitates the formation of cross-link C-C bonds. The disappearance of the acrylate carbon double bond (-C=C-) at peaks 1641, 1620, 1409, 981, and 817 cm^−1^ analysed using FTIR indicate that the APO nanoparticles have undergone cross-linking after the irradiation, as shown in the CTAB microemulsion system in Figure 12 [66]. These findings showed that the irradiation technique could be used to produce nanoparticles for usage in drug carriers of below than 100 nm particle size, as confirmed in TEM images presented in Figure 11.

Furthermore, Sadrolhosseini et al. reported on gold nanoparticles (Au-NPs) synthesised from palm oil using green and simple laser irradiation techniques [30,73]. Using the electron transfer from the carboxylic group to Au-NPs, the tail of the carbonyl band of palm oil was used as a capping agent for Au-NP. Gold nanoparticles with a particle size ranging from 8.92 to 19.73 nm were formed in the spherical shape of palm oil. These Au-NPs have a wide range of applications in drug delivery, microelectronics, environments, photonics, electronics, photodynamic therapy, therapeutic agent delivery, biosensors, sensors, medical diagnosis, and catalysis.

**Figure 13:**
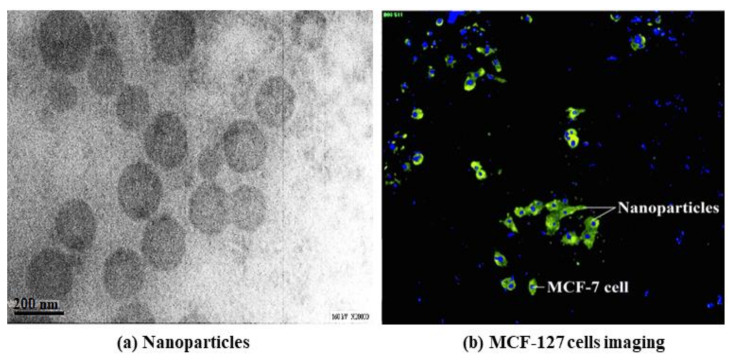
Peptide functionalised- and paclitaxel-loaded poly (APO-*b*-polyol ester) nanoparticles against MCF-7 cells. (**a**) Peptide was functionalised to poly(APO-*b*-polyol ester) nanoparticles using chemical conjugation technique and was loaded with paclitaxel; (**b**) Promising results were shown where the functionalised nanoparticles were found to be able to cause inhibition of human breast cancer cells. Source: unpublished data.

In recent a study by Tajau et al., gamma irradiation-induced RAFT was used for the development of targeted copolymer nanoparticles using APO and polyol ester as reactants of starter polymer matrices (Figure 13) [74]. The poly(APO-b-polyol ester) nanoparticles are made from palm oil products using the RAFT technique induced by gamma radiation. These copolymer nanoparticles are potentially used in drug delivery systems for breast cancer.

Reports also showed that various copolymers were developed using oleic acid (OA) by RAFT polymerization [75,76,77]. Previously, Zengin et al. developed hybrid silica-coated magnetic nanoparticles using oleic acid as a stabiliser via RAFT polymerization [75,76]. The surface-mediated RAFT polymerisation approach enables for synthesising less than 20 nm hybrid composite particles size. These particles have displayed superparamagnetic properties (saturation magnetisation = 35.4 emu g^−1^) and precise identification of molecular cholesterol. These nanoparticles could become a single magnetic domain at such a small scale and perform like a single super spin that shows strong magnetic susceptibility, enabling these nanoparticles to have a better and faster magnetic reaction relative to bulk magnets because under the control of an applied magnetic magnet, these superparamagnetic nanoparticles imprinted with cholesterol would contribute to the recovery of spiked human serum, milk, yolk, and beef for use in cell sorting, biomolecule enrichment and isolation, and drug delivery applications [76].

Meanwhile, a pH-responsive supramolecular graft copolymer micelle (Dextran-g-OA) composed of dextran and poly (oleic acid) was produced by Karmakar et al. via RAFT polymerization [77]. The Dextran-g-OA micelle of less than 300 nm in particle size demonstrated good loading efficacy and excellent release features to nifedipine for the oral delivery system. It also showed its cyto-compatible nature towards MG-63 cells for gastro-retentive drug delivery system. Nifedipine is used for the treatment of high blood pressure, cardiovascular disease, and chest pain relief. Comparatively, short gastric emptying time in humans may result in an incomplete release of Nifedipine from the existing drug delivery system that leads to a decreased effectiveness of the administered dose. As a result, this discovery could solve existing concerns to improve the effectiveness of nifedipine delivery to specific organs [78].

The RAFT polymerisation is a useful tool in the development of engineered polymeric materials due to its ability to perform at room temperature, aqueous media, and a controllable initiation process. However, the synthesis of copolymeric nanoparticles of the radiation-curable palm oil-based materials has yet to be widely reported primarily through radiation and RAFT techniques. Therefore, the usage of radiation and RAFT techniques for the development and modification of palm oil-based materials is also known to have a significant potential for drug delivery application. This review is functioned to explain further on this technique for producing palm oil-based copolymers nanoparticles for cancer therapy.

Besides, in the following section, it is highlighted that APO is not only a drug nanocarrier, but it also can be used in the production of biopolymeric scaffolding materials for use in medicine for the reconstruction and replacement of tissues and organs. Therefore, the use of APO in the production of drug delivery system and scaffolding is found necessary to replace existing synthetic medical devices due to safety and local cost concerns.

### 4.3. Scaffolds

**Figure 14:**
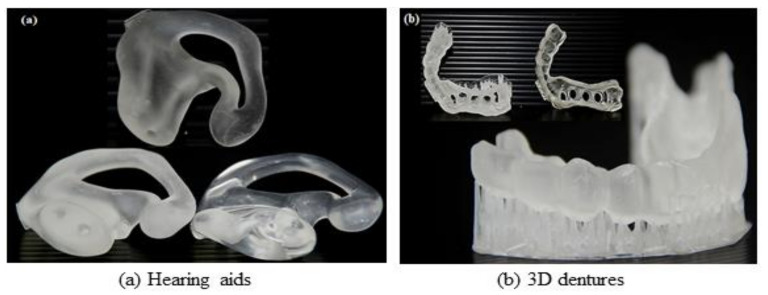
CAD model design using microstereolithography technique for (**a**) hearing aids and (**b**) 3D dentures. Source: Adapted from Tajau and Talib [80].

Radiation technologies play important roles in the development of tissue engineering, including the preparation and optimisation of instructive scaffolds. A new photopolymer resin prepared from APO resin has been formulated using a micro-stereo-lithography technique to be used in the manufacturing of tissue scaffold with a regulated microstructure and bioactivity, tailored porosity structure as well as inclusion phase shape and size [79]. The manufacturing of a tissue scaffold is carried out by an additive layer process in which the 3D object is sliced into a series of 2D layers with each of these layers produced until the 3D part is formed. Current calibration printing studies are performed using 100% poly(ethylene glycol) dimethacrylate (PEGDMA) with a photo-initiator present in each resin formulation using UV light. The appropriate design of the engineered tissue scaffold is carried out using CAD (computer-aided configuration) software. Figure 14 provides examples of scaffold products manufactured from APO using a micro-stereo-lithography technique. The generated tissue scaffolds, i.e., hearing aid and 3D denture, were predicted to have a controlled bioactivity due to starter scaffolding matrices made of radiation-curable palm-based materials that make it safe, biodegradable, and biocompatible as well as possess the desired porosity and mechanical properties [80].

Meanwhile, acrylic-based polymers, such as polyacrylic acid (PAA), are well known for their excellent biocompatibility and efficiency in mechanical properties. This has benefitted their usage for various significant biomedical applications, such as contact lenses, corneal prosthesis, bone cement, tissue engineering, nanofibres, nanocomposites, and nanoparticles [81]. Modification of polyacrylic acid using irradiation is especially useful for improving the properties of scaffolds based on acrylic. Radiation techniques have been reported as reliable methods for acrylic-based hydrogel synthesis for manufacturing of engineered tissue scaffolds [81]. For example, a cross-linked PAA hydrogel synthesised by cross-linking ionising radiation has been developed by Jabari et al. [82] and Rosiak et al. [27] for therapeutic dressing. Lee et al. also worked on UV irradiation method in interpenetration polymer network (IPN) hydrogels synthesis that composed of chitosan and poly(acrylic acid) (PAAc). A significant improvement has been shown in its mechanical properties, even in a hydrated condition [83]. Another reported study by Fabbri et al. also showed that the successful preparation of graphene oxide/acrylic resins through UV radiation of electrically conductive acrylic resins containing graphene oxide (rGO) has resulted in improved properties of conductive acrylic composites [84]. It can be said that almost all these studies revealed that when reacting to radiation, acrylic-based polymeric materials have a promising characteristic, as it continues to evolve as more sophisticated materials for tissue engineering products.

In a different study reported nearly a decade ago by Rao et al., they developed a poly(3-hydroxybutyrate-co-4-hydroxybutyrate), P(3HB-co-4HB) that was obtained by biosynthesis of *Cupriavidus necator* from spent palm oil [85]. Their findings showed that inexpensive spent palm oil is a good source of carbon in the processing of polyhydroxyalkanoates (PHA) efficiently using *Cupriavidus necator* via biosynthesis technique. This biopolymer ultimately can become a modern absorbable biomaterial for medical usage [86]. Kamilah et al. also supported their finding whereby they demonstrated that the palm oil-based waste cooking oil (PO-WCO) was found suitable as a sustainable source of carbon for cell growth and poly(3-hydroxybutyrate) P(3HB) biosynthesis [86]. However, radiation technique was not applied in both reported studies, thus has opened up the gap for utilising the technique in similar studies.

The production of radiation curable palm oil-based acrylic scaffolds through radiation processing could be carried out in no time in order to identify new strategies for improving and displaying superior biomaterial properties, especially for medical applications, as this technique is versatile and can be easily adapted. In addition to the materials being the starter matrices for polymeric scaffolds, as discussed in the following section, radiation-curable palm oil-based materials are also introduced in the use of polymeric composites as dental filling materials.

### 4.4. Dentistry

**Figure 15:**
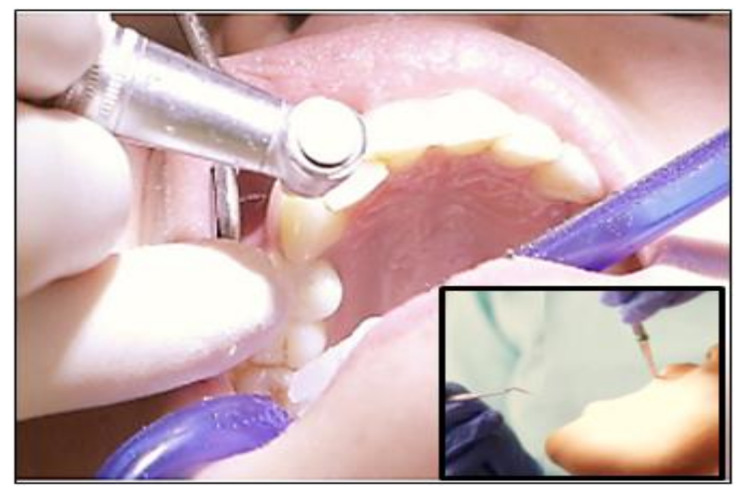
Ultraviolet light cure palm oil-based fillings.

A recent US patent granted in 2014 claimed on the polyurethane oligomer based on palm oil for usage as a dental restorative material [11]. Palm oil-based polyols possess more than two functional groups that induce branching of the polymer and tend to have higher crosslinking density as well as three-dimensional linked structures that provide greater mechanical strength and dimensional stability. The use of palm oil-based polyol can also improve the biocompatibility of the synthesised composition. The polyol and diisocyanate formulations have both soft and hard segments which are grafted by the polyol with isocyanate terminals with an unsaturated acrylic monomer to form a prepolymer with unsaturated terminals which is curable with free radical initiators. As a result, this can provide better dental filling materials that are fatigue resistance and have flexural properties. Figure 15 shows the UV light cure filling teeth dental process using curable materials, such as palm oil-based polyol dental filling materials. These composites of palm polyol were found susceptible to UV radiation, which were used as a coat for filling teeth cavities.

For several decades, acrylic-based resins with application in dentistry made up of polymethylmethacrylate (PMMA) or polyethylmethacrylate (PEMA) have dominated denture technology [87,88]. However, some of the drawbacks of these materials are the toxicity of the residual monomer, the complex wrapping procedure, complicated processing, and low resistance. Furthermore, there are also several different types of resin compounds, such as styrene, polycarbonate, polyethylene, polyethylene glycol, polydimethylsiloxane, poly(e-caprolactone), polypyrrole, hexamethyldisilazane, epiminic, polyurethane, vinyl, polyamide, acetal, and polyglass available for dental use in the manufacture of dentistry resins, most of which come from petrochemical-based polymer [87,89]. A recent study by Ionta et al. revealed that palm oil has gained superior protective capacity against tooth erosion, which appears to be a promising alternative for the prevention of initial erosion of enamel compared to coconut oil, safflower oil, sunflower oil, and olive oil [90]. Therefore, new resin alternatives, such as radiation-curable palm oil-based resins with a natural, renewable source, inexpensive, biodegradable, biocompatible, and promising physicochemical properties compared to petrochemical-based acrylics, are thus more suitable for dental applications.

Other features were reported in a few studies that were conducted on surface coating, overprint varnish (OPV), printing inks, pressure-sensitive adhesives (PSA), and dentistry for radiation-curable palm oil-based product applications [1,91]. These applications appeared to have a little exploration, but the palm oil-based products do have more possibilities to fine-tune the functionality of the radiation-curable products to satisfy the need for the application, including agricultural for seed treatments and coatings [92,93], restoration and consolidation of various cultural heritage artefacts as well as the use of radiation technology to disinfect cultural heritage artefacts [24,94,95].

## 5. Emerging Applications of Radiation Processing for Modification of Palm Oil-Based Polymeric Biomaterials for Potential Use in Biomedical Application

Radiation-modified palm oil-based materials is an example that has great potential in promoting new products for use in biomedical application, such as medical surface coating, medical device adhesives, smart drug delivery system, and tissue engineering devices. Besides, their favourable properties, such as natural properties, non-toxic materials, and cost-effective production lines due to the low cost of raw material can make palm oil-based products a good option for developing novel and advanced technology for use as polymeric biomaterials in the biomedical application.

### 5.1. Medical Surface Coating

The development of radiation modification of palm oil-based materials in advanced applications for superhydrophobic biomaterials, such as temporally implants, contact lenses, controlled drug-release coatings medical device coatings, and antimicrobial coatings could be of great promise. Several authors have reported potential development on radiation modification of palm-oil based material coating, such as acrylate, polyol, and urethane [40,47,96,97]. For instance, Said et al. used the combination of biopolymer of an acrylate palm oil-based EPOLA^®^/PETIA with different amount of SiO_2_ nanoparticles to produce good optical nanocomposites for numerous applications, including painting, sealants, adhesives, and coatings [54]. As a result, the increase of surface hardness and gel content values were achieved by increasing the silica loading in photocurable resin. In addition, the rapid decrease in water absorption in the presence of SiO_2_ allows such nanocomposites particularly desirable for coating applications. The nanocomposites comprising nanosilica distinguish exceptional barrier properties against moisture and gases as well as excellent resistance to corrosion [98]. Salleh et al. reported on producing hyperbranched curable polyurethane acrylate (HBPUA) made from palm oil oleic acid for applications in surface curing technology [40]. HBPUA is described as green and/or eco-friendly curable resin products with excellent thermal stability, especially upon subjecting to UV/EB irradiations.

Alias et al. introduced zirconium acrylate in EPOLA^®^ curable coating formulation in order to enhance EPOLA^®^ properties [99]. The presence of zirconium acrylate increases the adhesion and hardness of curable coating due to increment of the roughness and polarity of the surface. Meanwhile, the zirconium structure and properties contributed to the enhancement of film hardness. Mudri et al. also used EPOLA^®^ as a coating material on printed pages or known as overprint varnishes (OPV) [41]. The purpose of applying OPV is to enhance gloss, stain resistance, edge fusion resistance, resistance to burning and scuffing, and resistance to discolouration from environmental absorption of impurities. The EPOLA^®^-based OPV material possesses excellent properties for adhesion and glossiness.

Overall, the development of radiation-curable palm oil-based materials for surface coatings can reduce the risk of infection and prevent bacterial or microorganism attachment to the material surfaces. These materials must be chemically and physically non-reactive to the body as well as excellently comply when embedded in tissues. Therefore, the medical surface coating is necessary to reduce toxicity, side effects, and bacterial or microorganism infections without affecting public health.

### 5.2. Medical Light Curing Adhesives

Radiation processing of palm oil-based products could provide a wide variety of medical light curing adhesives for tape manufacture, pressure-sensitive adhesive (PSA), 3 M, and general adhesives. Radiation curable PSA was produced from different palm oil resins, such as EPOLA^®^ and EPOMA (epoxidised palm oil methacrylate). Such resins have found to improve coating, curing, and adhesive performances. Mahmood et al. studied the EPOLA^®^-PSA developed with various low-transition temperature acrylate and methacrylate monomers before curing with UV irradiation [100]. The findings showed that EPOLA^®^-based PSA exhibited fast curing speed as well as appropriate compatibility formulations, as demonstrated by their cured film properties, such as surface tackiness, peel adhesion, and creep resistance.

In 2017, Tugiman et al. produced UV curable maleinated acrylic epoxy palm oil PSA using UV LED system, which resulted in a small percentage of double conversion rate that was below 50% due to monomers and maybe even light transmission. This work is just a few known UV curable PSAs that have been reported for palm oils. The PSA synthesis of palm oil-based materials from the studies shows promising results in the production of radiation-curable palm oil-based PSA for medical device application [52].

### 5.3. Nanoscale Radionuclides and Radiolabelled Nanomaterial

Nanoscale radionuclides and radiolabelled nanomaterial for imaging, *diagnosis, and treatment of* APO and polyol ester had promising properties used by radiation-induced RAFT polymerisation and cross-linking techniques to produce targeted nanoparticles utilising radiation techniques. New features for the design of smart nanoparticles for nuclear molecular imaging and therapeutic purposes include the surface functionalisation of these nanoparticles with biomolecules, radioactive materials and also with a magnetic, plasmonic or fluorescent substance. The use of radiation modification in palm oil-based materials in nuclear nanomedicine in these latest technologies is expected to bring a paradigm shift in multimodal molecular imagery, thus facilitating a new era in medical imaging agents for diagnosis.

### 5.4. Three-Dimensional (3D) Scaffold for Tissue Engineering Application

The production of a photopolymer resin-based palm oil scaffold for bone tissue engineering therapy is a significant field for providing supply of biomaterials relating to the osseointegration of bioresorbable scaffolds and the control of bone infection. Talib et al. investigated the construction of a scaffold using materials based on palm oil, such as APO, using a micro stereolithography technique [79]. The study obtained favourable properties of dynamics, composition, bioactivity, porosity, and density. More innovation on the scaffolding of APO may display more substantial interests in potential applications, such as for tissue engineering. Meanwhile, potential physicochemical, mechanical, biodegradable, and biocompatibility properties were discovered in a water-blown porous polyurethane scaffold made of palm glycerol monostearate and glutaric acid, which demonstrated their tremendous biomaterial potential for soft and rigid tissue engineering applications [101,102].

Furthermore, a new soft biomaterial made from aliphatic polyurethane based on palm oil has recently been developed by Yeol et al. for tissue engineering applications. A polyester polyol (PPP) obtained from epoxidised palm olein and glutaric acid was used to prepare the polyurethane [103]. The polyurethanes displayed superior water absorption, >90% porosity, biodegradable, good thermal stability, and mechanical properties that made the generated polyurethanes able to be used for tissue repair and tissue regeneration applications.

In addition to the palm-based oleochemicals used for the development of biomaterials for tissue engineering, the palm oil mill effluent (POME) generated from palm oil mills and the palm oil empty fruit bunch (EFB) developed from palm oil extraction have also generated significant interest in the production of modern scaffold polyhydroxyalkanoates [104,105]. These studies have shown a promising hope that palm oil-based biomaterials can be used in a wide variety of tissue engineering applications. The different forms of biopolymers derived from palm-based oleochemicals and oil palm waste materials as biomaterials have significantly contributed to the manufacture of scaffolding products. Finally, radiation processing technology could be used to obtain more ideal characteristics of biomaterials for modifying the scaffolding products.

## 6. Way Forward

Finally, radiation-curable bio-based products from palm oil have shown great interest in biomedical applications for surface coating, adhesive, nanoscale radionuclides and radiolabelled nanomaterials, and scaffolding. For example, antimicrobial and anticorrosion properties can be added to the radiation curable polymeric materials for use in medical surface coatings for an active approach to killing microbial and surface protection and also for use in *dental* restorative in a wide variety of nanocomposites. It also can act as core platforms for medical radionuclide attachment and radiolabelled for molecular imaging and targeted drug delivery and as light cure adhesives, curable palm oil formulations that can be assembled as medical and surgical adhesive tapes for skin, wound dressing, and wound bandages. Meanwhile, 3D bioprinting can facilitate the development of functional tissue structures with radiation-curable polymeric based on palm oil approaches to mimic human tissues and organs. Therefore, new coatings like radiation-curable polymeric materials based on palm oil can benefit the production of medical devices. They are natural, enable the delivery of drugs, are surface-modified, and can be used to form artificial tissue. Radiation-curable products derived from palm oil have a bright future in the development of biomaterials since they originally come from sustainable resources are a convenient and environmentally friendly technology for the processing of raw materials and non-toxic products, and because of the availability of much cheaper products that make them comparable in terms of biocompatibility, biodegradability, durability, and the reasonability production cost compared to petrochemical-based products. Furthermore, radiation processing technology provides a simple, reliable, and environmentally safe manufacturing facility and is a powerful tool to induce physical and chemical reactions for use in the production of advanced modern materials.

## 7. Conclusions

As a conclusion, polymeric-based triglycerides and monomers, such as radiation-curable palm oil-based polymeric materials, have become alternatives to synthetic polymers. Thus, they are potentially useful for a diverse range of industries including biomedical applications such as surface coatings, drug delivery systems, and scaffolds. These polymeric materials are highly favourable for the above applications, as they are derived from natural resources, non-toxic, inexpensive, biocompatible, and biodegradable and possess promising thermal and mechanical properties. Moreover, the radiation-curable palm oil products obtained can be used as polymeric biomaterials in imaging, diagnostics and therapeutic purposes, medical surface coating and adhesives, radionuclides and radiolabelled nanomaterial, and tissue engineering. Therefore, the development of polymeric biomaterials based on radiation-induced palm oil fatty acids is found to be significant for broadening the use and application of palm oil in biomedical applications. Last but not least, the most considerable attraction and valuable insight in this invention is that the primary raw material comes from palm oil, which can be obtained easily from palm oil-producing countries.

## Figures and Tables

**Table 1:** The physicochemical properties of APO and polyol ester.

Type of Palm Oil-Based Products	APO [34]	Polyol Ester [32]
Acid value (mg KOH/g oil)	8.37	39.74
Saponification value (mg KOH/g oil)	221.89	297.00
Hydroxyl value (mg KOH/g oil)	46.41	182.51
Ester value	209.14	257.26
Viscosity (cps)	1774.67	1006.33
Molecular weight (Daltons)	1750.04	5004.00
Colour	Brownish	Dark

**Table 2:** The thermal and mechanical properties of the EPOLA^®^ and polyol-based products.

Developed Products	Thermal and Mechanical Properties	References
Acrylated resin from epoxidized oil (EPOLA^®^-based products)	Adhesion: 1.15 MPa Pencil hardness: H-2H	[51]
UV curable PSA of maleinated acrylated epoxidized palm oil (EPOLA^®^-based products)	Good and high adhesion properties	[52]
UV-curable polyurethane coating (EPOLA^®^-based products)	Adhesion: 0.5–1.5 MPa Pencil hardness: B and 2B Volatile organic compound: 5–15%	[53]
UV radiation-curable acrylic resin (EPOLA^®^-based products)	Degradation: 250–550 °C Pendulum hardness: 60% Tensile strength: 5.2–6.2 MPa	[42]
UV-curable acrylated coating (EPOLA^®^-based products)	Pendulum hardness: 55–75%	[43]
UV radiation-curable polyurethane resin (Polyol-based products)	Degradation: 200–455 °C Pencil hardness: B-2H	[48]
UV/EB-curable acrylated coating (EPOLA^®^-based products)	Scratch resistance: 0.1–0.9 N Pendulum hardness: 5–50% Pencil hardness: B-6H	[54]
UV curable acrylated polyol ester prepolymer (EPOLA^®^-based products)	Pendulum hardness: 49.4%	[55]

**Table 3:** Current development of palm oil-based products in drug delivery.

Product Development	Methods	Properties	Potential Application	References
Palm oil-based paracetamol suppositories	Double casting	More than 95% drug release	Oral delivery	[59]
Transparent microemulsion from palm oil-derived isopropyl palmitate (IPP) with Tween 80 and 1- butanol	Microemulsion	Particle size <150 nm pH: 6.76 to 7.80 Charge: −32 to −75 mV Storage stability for 4 weeks.	Transdermal and Topical	[60]
Palm oil based-organogels and microemulsions from palm oil, span 80/tween 80 mixture (organogel) and water	Microemulsion	Highly haemo-compatible Non-irritant	Topical delivery	[61]
Palm oil and soy oil-based organogels	Microemulsion	Good spreadability and vscosity profile highly biocompatible	Topical delivery	[62]
Palm Oil Esters (POEs)-based nanoemulsions	Microemulsion	Less than 500 nm	Topical delivery	[63]
Palm oil esters (POEs)-based nano-cream	Microemulsion	Less than 130 nm −20 mV Stable	Topical delivery	[64]
Microspheres encapsulating terbutaline sulphate nanoparticles using hydrogenated palm oil	Emulsion	3.9 um	Pulmonary delivery	[65]
Palm oil-based nanoparticle	Microemulsion and ionizing radiation	Particle size: 70–220 nm	Preliminary study for oral delivery	[66]
Solid lipid nanoparticle (SLN) using varieties of emulsifier of palm oil (S154) and lecithin (Lipoid 100)	High pressure homogenization (HPH)	Particle size: 140–300 nm Zeta potential of about −13 and −20 mV uniform size distribution	Colloidal drug delivery	[67]
Microcomposites theophylline/hydrogenated palm oil	Particles from gas saturated solutions (PGSS)	Mean particle size: 2.5–3.0 um Contain initially from 0.5 to 3.5% (weight) of theophylline uniform size distribution	Controlled drug delivery systems	[68]
Palm oil-based Liposomal Dox (Doxorubicin hydrochloride)	Freeze-thaw	(a) Distribution sizes of 438 and 453 nm (b) Zeta potential of about −31 and −32 mV Stability	Breast cancer delivery	[69]
Palm oil-based liposome	Thin film hydration	(a) Mean particle size: 340–450 nm (b) Zeta potential: −26 to −33 mV (c) Stable Biodegradability	Drug delivery device	[70]
Palm-oil-based Liposomal Dox (Doxorubicin hydrochloride)	Freeze-thaw	Distribution sizes of 438 and 453 nm Zeta potential of about −31 and −32 mV Stability	Breast cancer delivery	[71]
Palm oil-based liposome	Thin film hydration	Mean particle size: 340–450 nm Zeta potential: −26 to −33 mV Stable Biodegradability	Drug delivery device	[72]
Silver nanoparticles	Laser ablation	2.5–2 nm	Nanocomposite fabrications and antibacterial	[71]
Gold nanoparticles	Laser ablation	8–20 nm	Drug delivery device	[30]

## Data Availability

Not applicable.
